# Supplementary figures and images for: Immunomodulation of the Innate Host Response by Mesenchymal-Derived Versican during Influenza A Virus Infection
Source: bioRxiv. 2025 Aug 2:2025.08.01.668194. Preprint. [Version 1] doi: 10.1101/2025.08.01.668194 (PMC12324535; doi:10.1101/2025.08.01.668194)

Supplemental Figure S1.

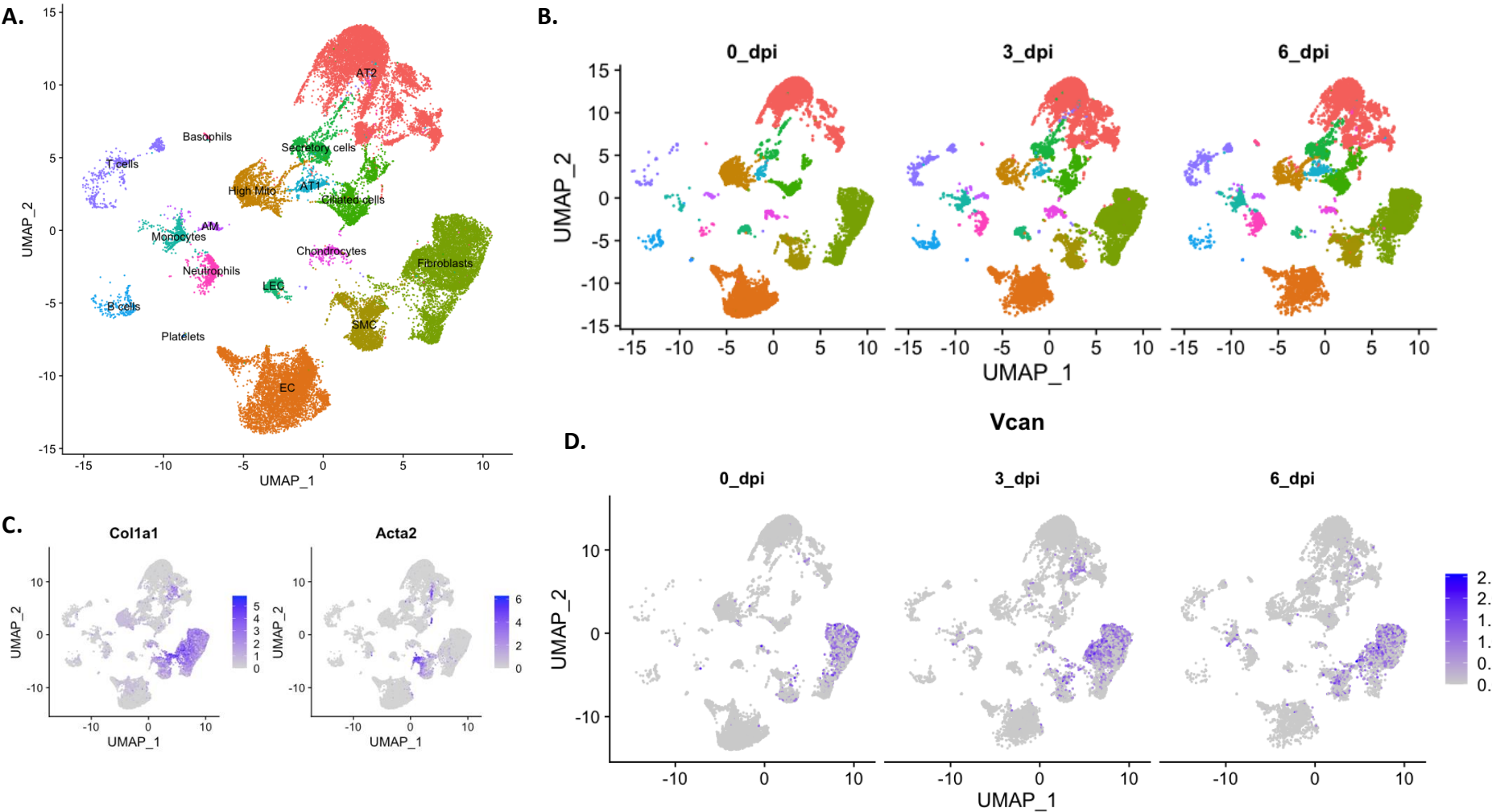

Supplemental Figure S2.

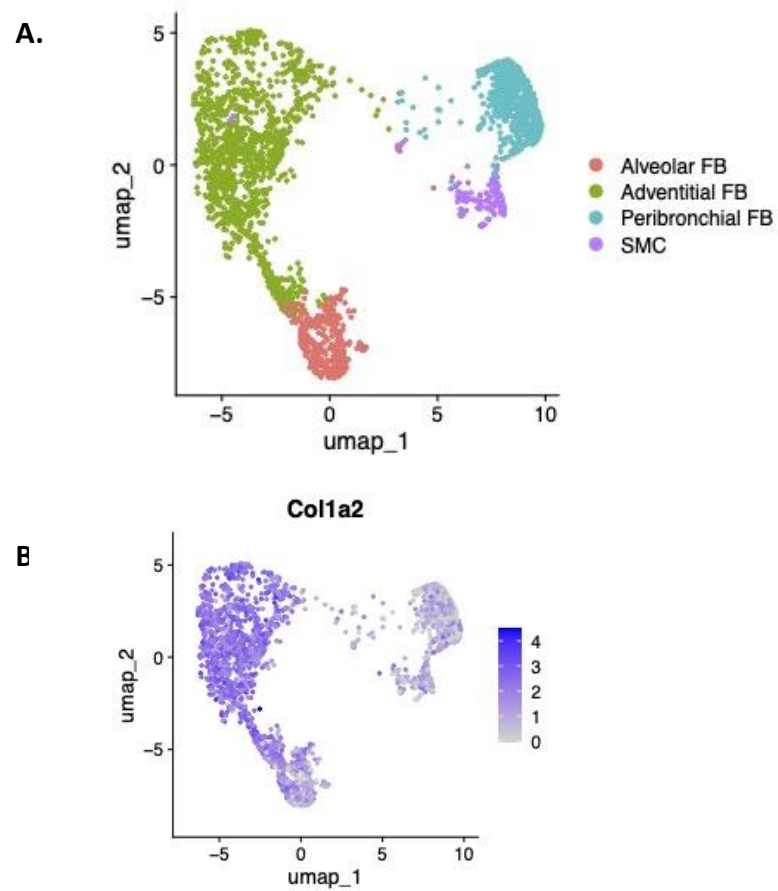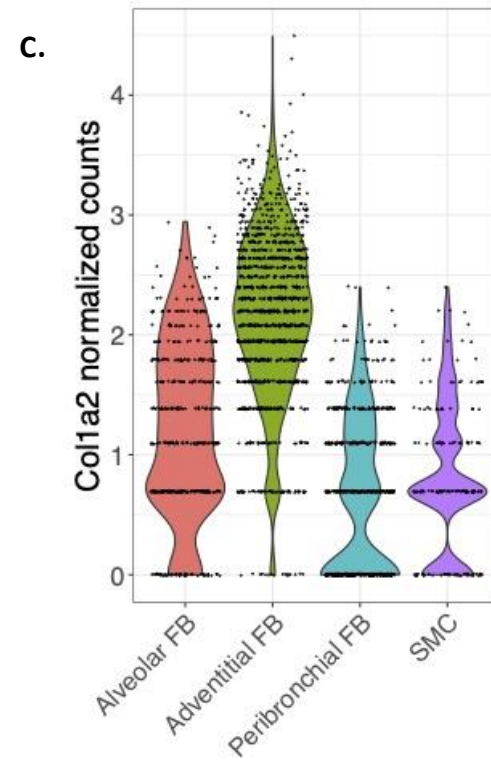

**Supplemental Figure S3.**

**A.**

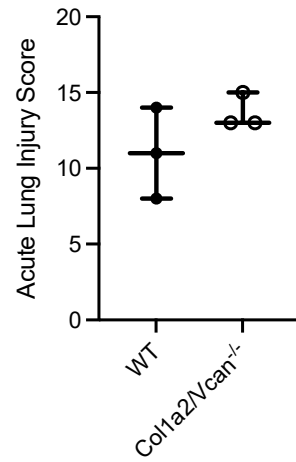

**B.**

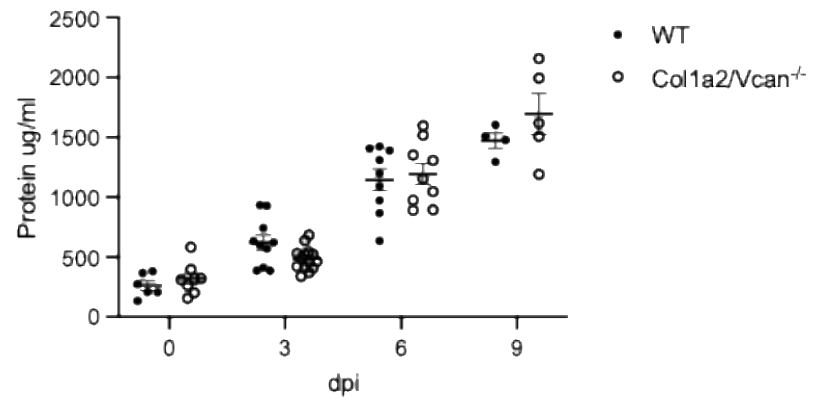

**Supplemental Figure S4.**

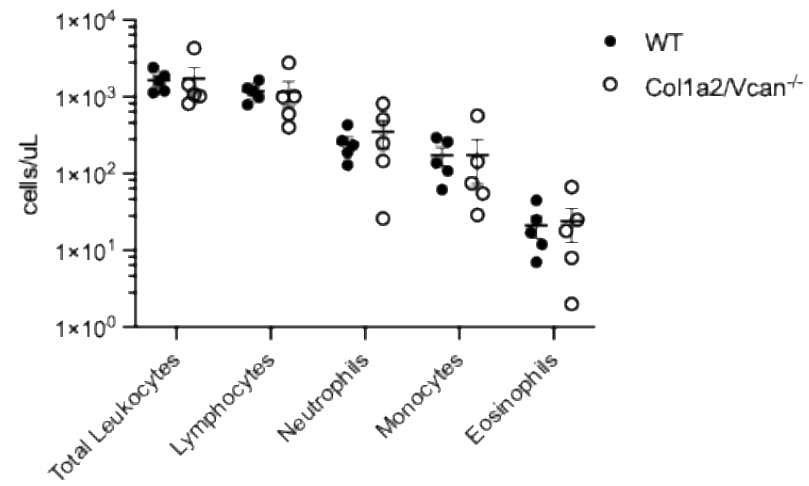

Supplement: Supplement 1 — Supplemental Figure S1. UMAP visualization of lung cells and versican expression of mice on days 0-, 3-, and 6-dpi with IAV. (A) Uniform manifold approximation and projection (UMAP) plot of the scRNAseq data from uninfected mice. (B) Cell clusters of different colors represent the various populations of cells in the lungs 0, 3, and 6-dpi with IAV. (C) UMAP visualization of Col1a1 and Acta2 normalized counts. (D) UMAP visualization of Vcan normalized counts (purple dots) in all cells of the lungs on 0, 3, and 6-dpi with IAV. Grey illustrates cells not expressing Vcan mRNA. Abbreviations: AT1, alveolar type 1; AT2, alveolar type 2; AM, alveolar macrophages; LEC, lymphatic endothelial cells; EC, endothelial cells Supplemental Figure S2. Col1a2 expression in mesenchymal cells from whole lungs of mice 0-dpi with IAV. (A) Uniform manifold approximation and projection (UMAP) visualization of mesenchymal cell clusters recovered from whole lungs of mice. (B) Col1a2 normalized counts in the four clusters of mesenchymal cells in control mice (0 dpi) (C) Col1a2 expression in the four clusters of mesenchymal cells in control mice (0 dpi). n=4=5 mice per group. Supplemental Figure S3. Histological evidence of tissue injury and alteration of the alveolar-capillary barrier during IAV (A) Acute lung injury scores from WT and Col1a2/Vcan−/− mice 9 dpi with IAV. (B) Total protein measured from BAL fluid at 0 (PBS), 3, 6, and 9 dpi with IAV. Values are mean ± SEM, n = 3 in (A) and n = 4–14 in (B). Mann-Whitney for (A) and two-way ANOVA with Bonferroni’s for multiple comparison test for (B). Abbreviations: BAL, bronchoalveolar lavage Supplemental Figure S4. Circulating Leukocytes on 3 dpi with IAV Total leukocytes, lymphocytes, neutrophils, monocytes, and eosinophils in circulation was determined from whole blood samples collected from Col1a2/Vcan−/− and WT mice 3 dpi with IAV. No differences were observed between Col1a2/Vcan−/− and WT mice using Student’s t-test. Values are mean ± SEM, [file media-1.pdf]
